# Converse magneto-electric effects in a core–shell multiferroic nanofiber by electric field tuning of ferromagnetic resonance

**DOI:** 10.1038/s41598-020-77041-x

**Published:** 2020-11-19

**Authors:** Ying Liu, G. Sreenivasulu, P. Zhou, J. Fu, D. Filippov, W. Zhang, T. Zhou, T. Zhang, Piyush Shah, M. R. Page, Gopalan Srinivasan, S. Berweger, T. M. Wallis, P. Kabos

**Affiliations:** 1 Department of Physics, Oakland University, Rochester, MI 48309 USA; 2 Department of Materials Science and Engineering, Hubei University, Wuhan, 430062 China; 3 Department of Materials Science and Engineering, Virginia Tech, Blacksburg, VA 24060 USA; 4 College of Electronics and Information, Hangzhou Dianzi University, Hangzhou, 310018 China; 5 Yaroslav-the-Wise Novgorod State University, Veliky Novgorod, Russia; 6 Materials and Manufacturing Directorate, Air Force Research Laboratory, Wright-Patterson Air Force Base, Dayton, OH 45433 USA; 7 Applied Physics Division, National Institute of Standards and Technology, Boulder, CO 80305 USA

**Keywords:** Materials science, Nanoscience and technology, Physics

## Abstract

This report is on studies directed at the nature of magneto-electric (ME) coupling by ferromagnetic resonance (FMR) under an electric field in a coaxial nanofiber of nickel ferrite (NFO) and lead zirconate titanate (PZT). Fibers with ferrite cores and PZT shells were prepared by electrospinning. The core–shell structure of annealed fibers was confirmed by electron- and scanning probe microscopy. For studies on converse ME effects, i.e., the magnetic response of the fibers to an applied electric field, FMR measurements were done on a single fiber with a near-field scanning microwave microscope (NSMM) at 5–10 GHz by obtaining profiles of both amplitude and phase of the complex scattering parameter *S*_*11*_ as a function of bias magnetic field. The strength of the voltage-ME coupling *A*_*v*_ was determined from the shift in the resonance field *H*_*r*_ for bias voltage of *V* = 0–7 V applied to the fiber. The coefficient *A*_*v*_ for the NFO core/PZT shell structure was estimated to be − 1.92 kA/Vm (− 24 Oe/V). A model was developed for the converse ME effects in the fibers and the theoretical estimates are in good agreement with the data.

## Introduction

Recently there has been an intense effort in the investigation of micro/nanostructures of heterogeneous materials and configurations. Among those there is considerable interest in multiferroic composite nanostructures^1–8^. These materials have several different configurations depending on intended application and include patterned nanostructured analogs of multiferroic materials^9^. One such composite with ferromagnetic and ferroelectric phases is of particular importance for studies on magneto-electric (ME) coupling between the ferroic phases facilitated by mechanical strain^4,5^. The coupling occurs through magnetostriction in the ferromagnetic phase, which in turn results in an electrical response due to piezoelectric effect in the ferroelectric phase. Nano-composites with high surface area-to-volume ratio are of specific interest due to predictions of strong ME coupling^10^. Nano-composites studied in recent years include core–shell particles^11–13^, ordered arrays^14–17^, nanobilayers^18–24^, nanopillars in a host matrix^23,24^, and coaxial fibers^25–36^. Nanofibers, however, have the potential for achieving a strong ME coupling due to the absence of substrate clamping encountered in bilayers or nanopillars on substrates^9^. Materials with strong ME coupling offer several unique application possibilities including dual electric and magnetic field tunable signal processing devices, ultrasensitive magnetic sensors, and applications in energy harvesting and information storage technologies^3–6^. Some of the specific device applications for the fiber composites are microwave absorbers, medical, acoustical, optical, magneto-optical and spintronic devices, and data storage applications^7^.

Here our focus is on ferrite-ferroelectric core–shell nanofibers. There have been some reports in the past on the direct- and converse magneto-electric effects for several ferromagnetic and ferroelectric core–shell fibers^25,36^. For direct ME (DME) effects, one measures the influence of an applied magnetic field *H* on ferroelectric order parameters^26–28^. The Converse ME (CME) effects are studied by applying an electric field and measuring the resulting variation in magnetic order parameters^9^. Efforts so far focused primarily on ME measurements on fibers assembled into a thin film in a magnetic field or fibers pressed into pellets ^25–29^. The key challenge in the case of multiferroic nanofibers, however, is the determination of ME coupling strength with measurements on an individual nanofiber. One of the methods used in the past was to measure the DME coupling by piezo-response force microscopy (PFM) under an applied *H* field ^37^. The ME coupling in CoFe_2_O_4_–Pb(Zr_0.52_Ti_0.48_)O_3_ core–shell nanofibers measured by PFM was reported to be quite strong compared to bulk or layered composites^38^. The strain-mediated coupling between CoFe_2_O_4_ and piezoelectric polyvinylidene fluoride was reported to show an increased piezoelectric coupling coefficient under an applied *H* field^33^. The strength of CME coupling is measured either by magnetization *M* vs *H* in the presence of an electric field *E* or ferromagnetic resonance (FMR) under *E* ^3,21^. There have no reports so far on CME effects in an individual ferrite-ferroelectric nanostructure.

In this work, we report on the CME effects in a coaxial fiber of nickel ferrite (NFO) and lead zirconate titanate (PZT) prepared by electrospinning. The core–shell structures were confirmed using scanning electron microscopy (SEM) and near-field scanning microwave microscopy (NSMM) The strength of CME coupling was measured by performing local FMR measurements under an applied *E* field with an NSMM in fibers with ferrite core-PZT shell. Data on the shift in the resonance field *δH*_*r*_ at 5–10 GHz were obtained as a function of applied DC voltage *V* for estimating the coupling coefficient *A*_*v*_ = *δH*_*r*_* /V* and was determined to be − 1.92 kA/m (− 24 Oe/V) for fibers of NFO core-PZT shell. We developed a theory for the CME effect in the fibers and the estimated *A*_*v*_-values are in good agreement with the measured values.

## Results

### Characterization of the core and shell fibers

**Figure 1:**
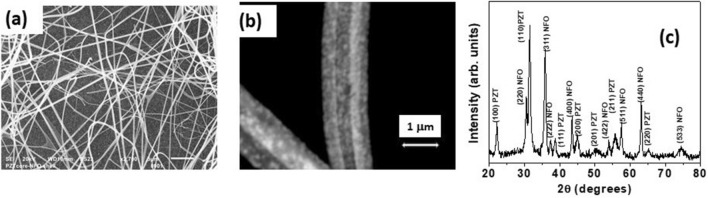
(**a**) SEM micrograph of NFO core-PZT shell fibers. (**b**) SEM image of a single fiber of NFO core and PZT shell. (**c**) XRD data for annealed fibers of NFO core-PZT shell.

Figure 1a shows the SEM micrograph for a collection of fibers with NFO core-PZT shell. Uniform fibers of diameter 400–1200 nm and length of 10–30 μm are seen. Figure 1b shows the SEM micrograph for a single fiber. The fiber is not a continuous medium. It is clear from the image that the core and shell are in fact nanocrystallites of NFO and PZT of unknown orientation. The core and shell in the sample are well resolved and the image shows a fiber with 400 nm diameter NFO core and 400 nm thick PZT shell with an estimated core-to-total volume ratio of 0.11. We verified the phase purity both NFO and PZT in the fibers by X-ray diffraction (XRD). Figure 1c shows the XRD data for annealed fibers and the diffraction peaks correspond to either NFO or PZT. The ferrite and PZT compositions were confirmed with energy dispersive X-ray spectroscopy measurements and the estimated compositions are Pb Zr_0.6_Ti_0.4_ O_3_ that is preferred for its large piezoelectric coupling coefficient and NiFe_2_O_4_.

**Figure 2:**
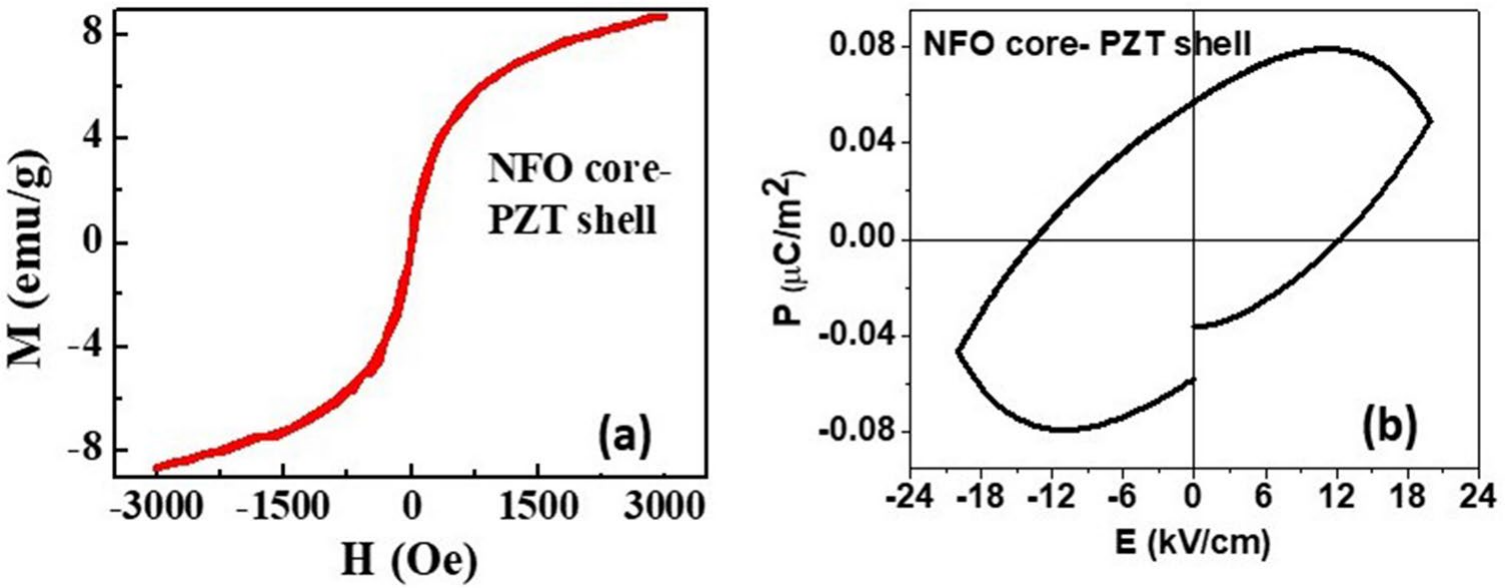
(**a**) The room temperature magnetization *M* vs magnetic field *H* for an ensemble of NFO core-PZT shell nanowires. (**b**) The polarization *P* vs electric field *E* for NFO core-PZT shell measured on a disc shaped annealed pellet of the fibers.

Results on static magnetization and ferroelectric polarization measurements for ensembles of these composite fibers are shown in Fig. 2. In order to measure the average ferroic order parameters for the fibers, we made discs with 5 mm in diameter and 0.5 mm in thickness by pressing the fibers in a die. This was followed by high temperature annealing. The annealed fiber discs were then used for measurements of ferroelectric polarization. Figure 2a shows the room temperature magnetization *M* as a function of static magnetic field *H* for annealed fibers and Fig. 2b show the ferroelectric polarization *P* as a function of electric field *E* measured on disc shaped pellet of annealed fibers. The magnetic hysteresis loop in Fig. 2a was measured for *H* up to 240 kA/m (3 kOe) and shows a maximum *M*-value of 9 Am^2^/kg (9 emu/g). The magnetization is not saturated under the maximum applied field of 240 kA/m (3 kOe). The *M* value is rather small compared to 44 Am^2^/kg (44 emu/g) for bulk NFO^40^, which can be attributed to a relatively small weight (or volume) fraction for the ferrite in the fiber. Our SEM measurements showed a distribution in the fiber diameter from 400 to 1200 nm with 60% of fibers with diameter 800–1000 nm. The volume of the ferrite in the fibers varied from 10 to 30% of the total volume. The ferroelectric nature for core–shell fibers were demonstrated by measurements of *P* vs *E* in Fig. 2b. The remnant polarization is 0.056 μC/cm^2^ and is two orders of magnitude smaller than for pure PZT fibers^41^. The low polarization may be attributed to a large leakage current due to the ferrite core. The fiber pellet is essentially a bulk composite with a low resistivity ferrite giving rise to a large leakage current as is evident from Fig. 2b.

### Converse ME effects on core–shell fiber by FMR

**Figure 3:**
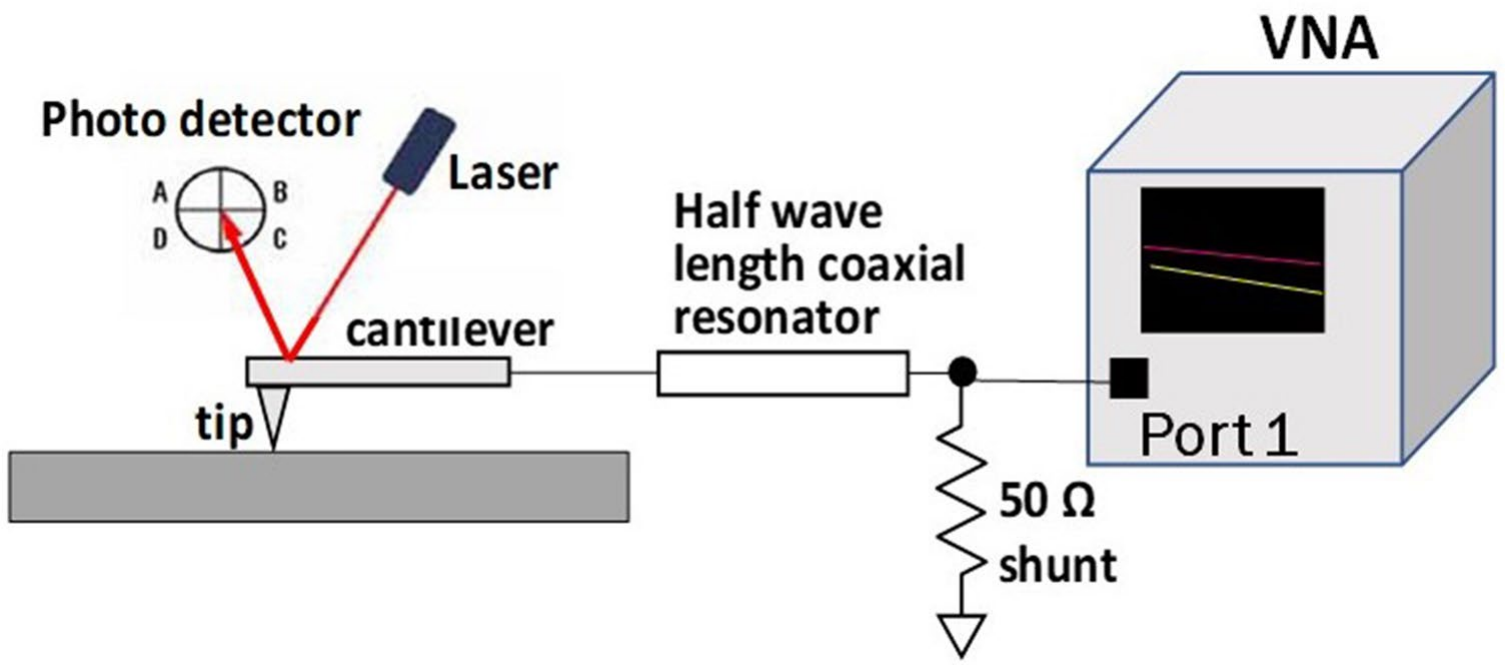
Schematic diagram showing the set up for ferromagnetic resonance measurements on the fiber using a near field scanning microwave microscope.

Local FMR measurements were done with a commercial broadband near field scanning microwave microscope (NSMM)^42,43^ modified for magnetic measurements. A schematic of the measurement configuration is shown in Fig. 3. NSMM is essentially an atomic force microscope (AFM) with added broadband frequency measurement capability. The main functions of the microscope are those of a standard AFM allowing nanometer-scale spatial resolution. In addition to conventional AFM topographic imaging capabilities the system has a modified scanner that allows transmission of a signal with frequencies between 2 and 20 GHz to the tip of the cantilever. Typically, the measurand is the reflection coefficient $S_{11} = (Z - Z_{0} )/\left( {Z + Z_{0} } \right)$, where *Z* is the complex impedance at the tip of the cantilever and $Z_{0}$ is the characteristic impedance of the microwave system. The reference impedance is usually selected to be 50 Ω in high frequency test equipment. For our purpose, both the amplitude and phase of the reflection coefficient is measured with the tip in contact with the nanowire, as a function of the frequency, bias voltage and/or applied fields.

**Figure 4:**
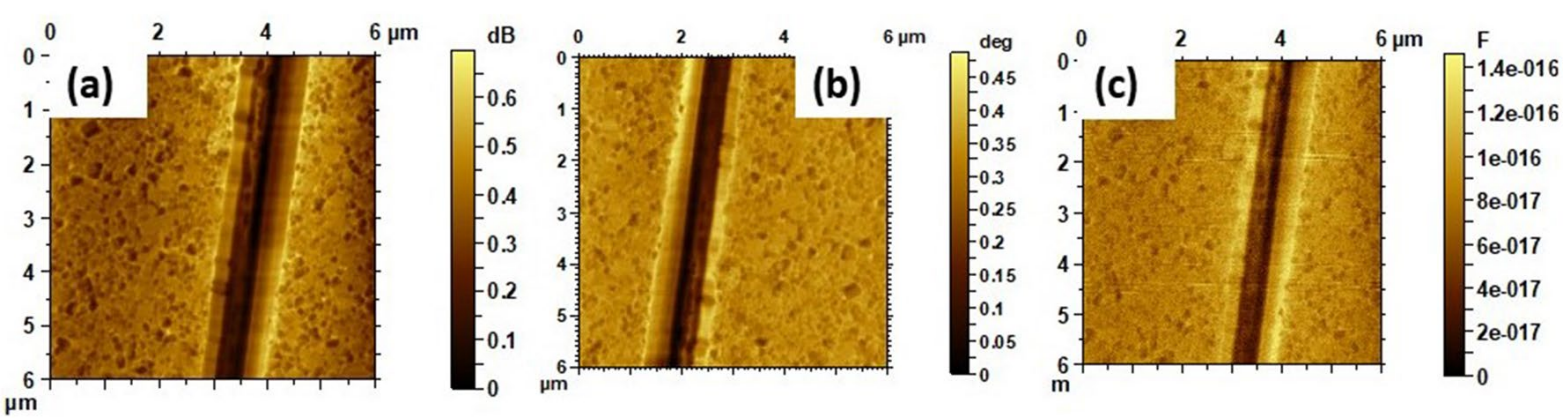
NSMM images of (**a**) S_11_ amplitude, (**b**) S_11_ phase, and (**c**) capacitance at 5.4 GHz for a single NFO core-PZT shell fiber.

Usually a vector network analyzer (VNA) is used as a source of microwave power and for the measurement of the reflection coefficient *S*_*11*_. The impedance at the tip-sample interface is a function of the local material parameters and as such is used to de-embed those properties. Figure 4 shows the *S*_*11*_ amplitude, *S*_*11*_ phase, and capacitance images of a single NFO core-PZT shell nanofiber at 5.4 GHz. The images illustrate the capability of this technique. Shell and the core of the nanofiber can be clearly distinguished in the *S*_*11*_ phase image, although they show up also less pronounced in the topography and *S*_*11*_ amplitude images. They corroborate the structural information obtained from SEM imaging.

**Figure 5:**
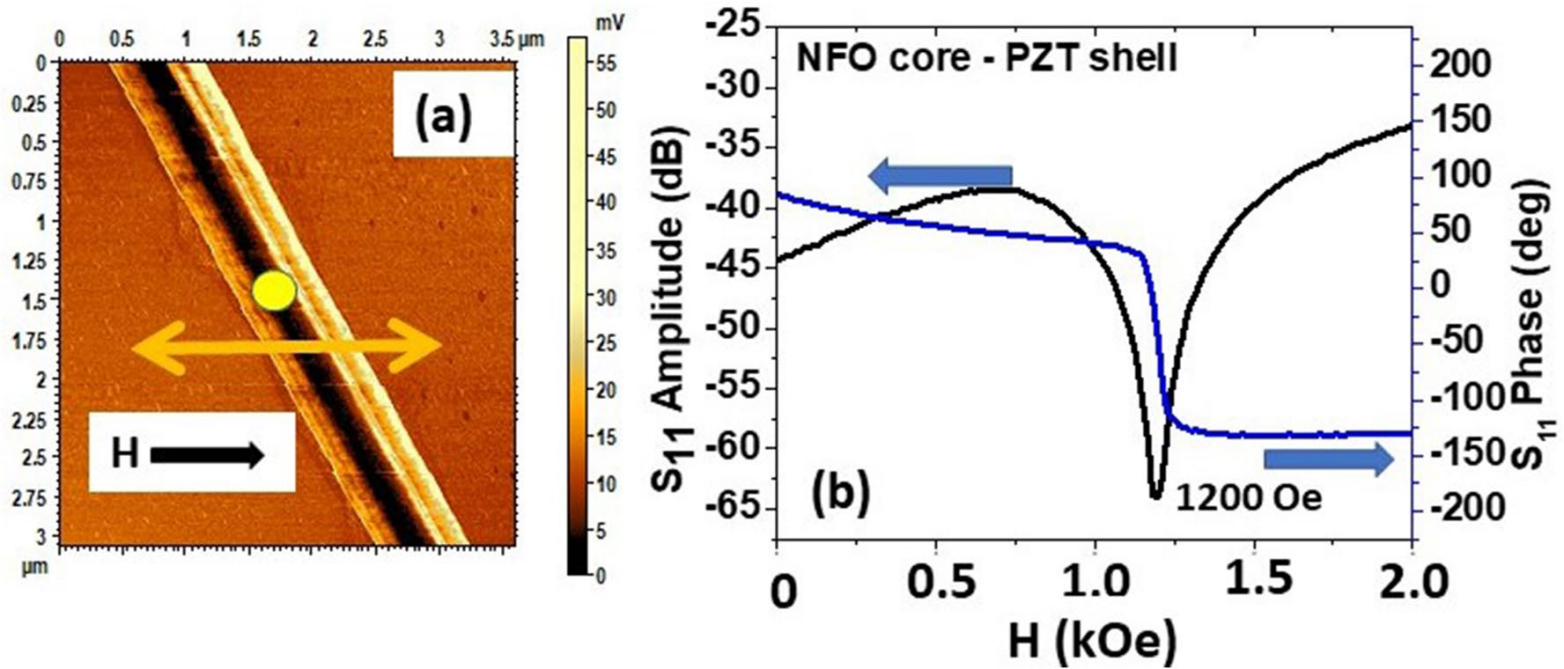
(**a**) NSMM S_11_ image of single NFO core-PZT shell fiber used for FMR measurements under an applied magnetic field *H*. The yellow dot represents the position of the NSMM tip during the measurements. The total nanowire diameter is 800 nm and the NFO core diameter is 400 nm. (**b**) S_11_ amplitude and phase vs applied magnetic field *H* at 5.4 GHz for NFO core-PZT shell single fiber.

As mentioned above the NSMM can measure the frequency-dependent response of a single fiber specimen as a function of different external stimuli. Samples for measurements of FMR required a special procedure as described below. A few mg of annealed fibers were first mixed with 10 mL of ethyl alcohol. A few drops of sonicated fiber-alcohol solution was placed on a glass slide placed between the pole pieces of a permanent magnet that produced a magnetic field of 1600 kA/m. For the measurement of FMR in NFO/PZT nanofibers we used a modified NSMM with field scan option. The magnetic field in the modified system is delivered by an electromagnet with + 1600 to − 1600 kA/m field sweep capability in a 2 mm wide gap. The measurement was done at several frequencies. At each frequency the magnetic field was continuously scanned between 0 and + 160 kA/m. The field was applied obliquely with respect to the axis of the nanowire as shown in Fig. 5a. During the field sweep, the reflected microwave signal from the nanowire was recorded by a vector network analyzer as a function of the magnetic field and for a series of tip DC voltage biases. The yellow circle in Fig. 5a shows the position of the tip during magnetic field sweep and Fig. 5b shows examples of the recorded *S*_*11*_ vs *H* profile for NFO core-PZT shell nanofiber at 5.4 GHz. The *H*-value (96 kA/m, 1200 Oe) corresponding to the dip in the amplitude and 180° phase change, both occurring at the same bias field, represents the FMR resonance field for the NFO core in the nanofiber. NSMM data acquired by use of the experimental procedure described above were analyzed to determine CME effects. This analysis was done in two steps. The FMR profiles were first obtained as a function of frequency *f* and data on resonance field *H*_*r*_ vs *f* were used to estimate the magnetic parameter for the fiber. This was followed by measurements of *H*_*r*_ as a function of the DC voltage *V* applied to the fiber for a specific excitation frequency. Data on *H*_*r*_ vs *V* were then used for the estimation of the voltage-CME coupling coefficient *A*_*v*_.

**Figure 6:**
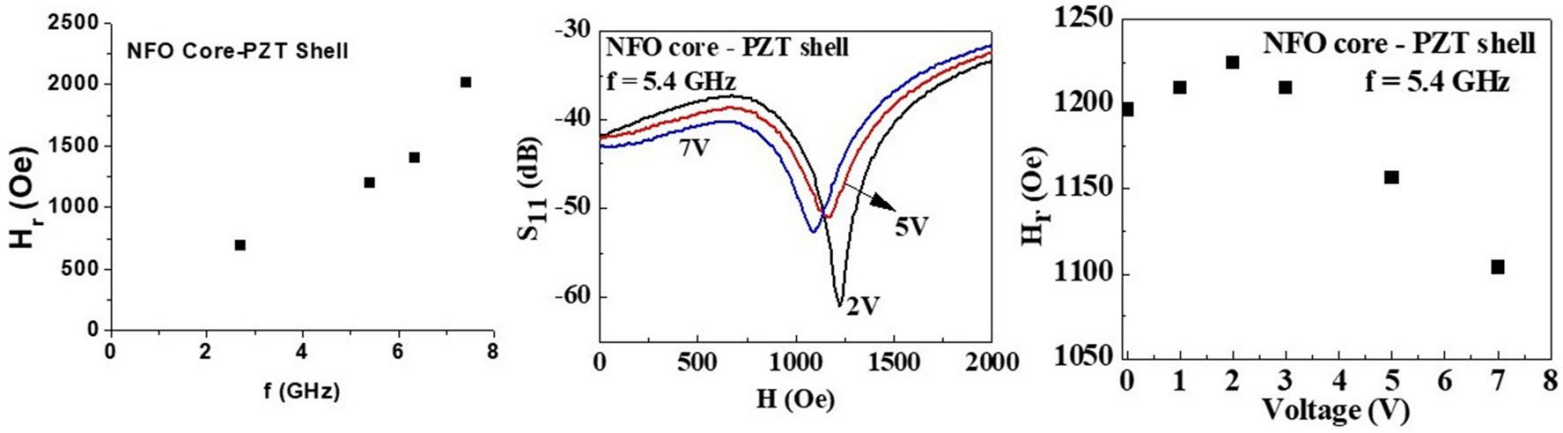
FMR signal measured with NSMM for NFO core-PZT shell fiber, (**a**) FMR resonance field *H*_*r*_ vs frequency f. (**b**) S_11_ amplitude vs magnetic field *H* with different applied voltage at 5.4 GHz. (**c**) The FMR resonance field *H*_*r*_ vs applied voltage of 0–7 V at 5.4 GHz.

Next, we consider resonance measurements on a fiber with an NFO core and a PZT shell. Figure 6a shows *H*_*r*_ vs *f* for *f* = 2.6–7.5 GHz that were obtained from FMR profiles such as the one in Fig. 5b. The increase in *H*_*r*_ with *f* is evident from the data, which can subsequently be analyzed to estimate the magnetic parameters for the nanowire. In our case the applied field *H* is at an angle *θ*_*H*_ = 45° to the axis of fiber and we use the resonance condition obtained in a recent study on FMR in core–shell fibers of yttrium iron garnet and a ferroelectric^29^. For non-interacting fibers and for *θ*_*H*_ = 45° one obtains:

1 $$\frac{f}{\gamma } = \frac{{H_{r} }}{\sqrt 2 } + H_{eff}$$

where γ is the gyromagnetic ratio and *H*_*eff*_ is the effective field which is the sum of crystalline anisotropy *H*_*a*_ and demagnetizing field *M*_*s*_ (*4πM*_*s*_). Assuming γ ~ 30 GHz/T for the NFO core^40^ and using the data in Fig. 6a one obtains an average value of *H*_*eff*_ = 72 kA/m (0.9 kOe). For the fiber in Fig. 5a with a total diameter of 800 nm and NFO core diameter of 400 nm, the approximate ferrite volume is 30% of the total volume. Using the bulk ferrite-only value of *M*_*s*_ = 240 kA/m (*4πM*_*s*_ = 3 kG), *H*_*eff*_ = 72 kA/m (0.9 kOe) is estimated from FMR data, which indicates negligible crystalline anisotropy in the ferrite core of the fiber.

The interpretation of the FMR linewidth data in Figs. 5 and 6 is complicated for the presented measurements because in NSMM measurements the excitation RF field is highly nonuniform leading to excitation of multiple modes. Furthermore, the measurement is in the near-field regime, which leads to excitation of modes in a broad wavevector range. The situation is even more complicated due to oblique orientation of the applied static magnetic field with respect to the nanofiber axis. To simplify the analysis, we are focusing only on the influence of the frequency and tip electrical bias on the resonance field. These effects are also the most relevant for many potential applications of the composite multiferroic nanofibers.

For the determination of the strength of the CME interactions, resonance measurements were done at a fixed frequency and for a series of DC voltage applied to the fiber. A tip-bias voltage in the range *V* = 0–7 V were applied to the PZT shell that correspond to a maximum electric field of *E* ~ 11 MV/m. Figure 6b shows the FMR profiles for representative voltages and the dips in the S_11_ amplitude represent the local FMR response of the NFO core of the fiber. The profiles in Fig. 6b were obtained at 5.4 GHz. The FMR is down-shifted to lower magnetic field [left in Fig. 6b] as the voltage is increases from 2 to 7 V. Figure 6c shows the FMR resonance field *H*_*r*_ vs *V* at 5.4 GHz, with the value of *H*_*r*_ increasing initially to 98 kA/m (1225 Oe) at *V* = 2 V, and then decreasing approximately linearly with *V* to 88 kA/m (1100 Oe) for applied voltage of 7 V. From these data, the estimated voltage-CME coupling coefficient *A*_*V*_ = *dH*_*r*_*/dV* = − 1.92 kA/Vm (− 24 Oe/V). The corresponding CME coefficient *A*_*E*_ = *dH*_*r*_*/dE* = − 1.6 kA/MV (− 20 Oe m/MV).

Next we develop a model for the CME effects in the nanofiber and compare the strength of CME interaction in the nanofiber with values reported for thin films and layered ferromagnetic-ferroelectric composites.

## Theory and discussion

Here we consider a model for the CME interactions in the coaxial fiber with a magnetostrictive core and piezoelectric shell. The shift in FMR under an electric field occurs as a result of interaction between the magnetic and piezoelectric phases that is aided by the mechanical deformation. The piezoelectric strain under *E* is transmitted through the interface to the magnetostrictive phase and the resulting magnetoelastic interactions manifest as a change in the effective magnetic field and a corresponding shift of the resonance line. This contribution to the effective magnetic field depends on the mechanical stress in the magnetic phase, magnetostriction constant and the saturation magnetization^44,45^. For the determination of the stress component we used the following method based on the solution of the elastic equation system for the magnetostrictive and piezoelectric phases.

**Figure 7:**
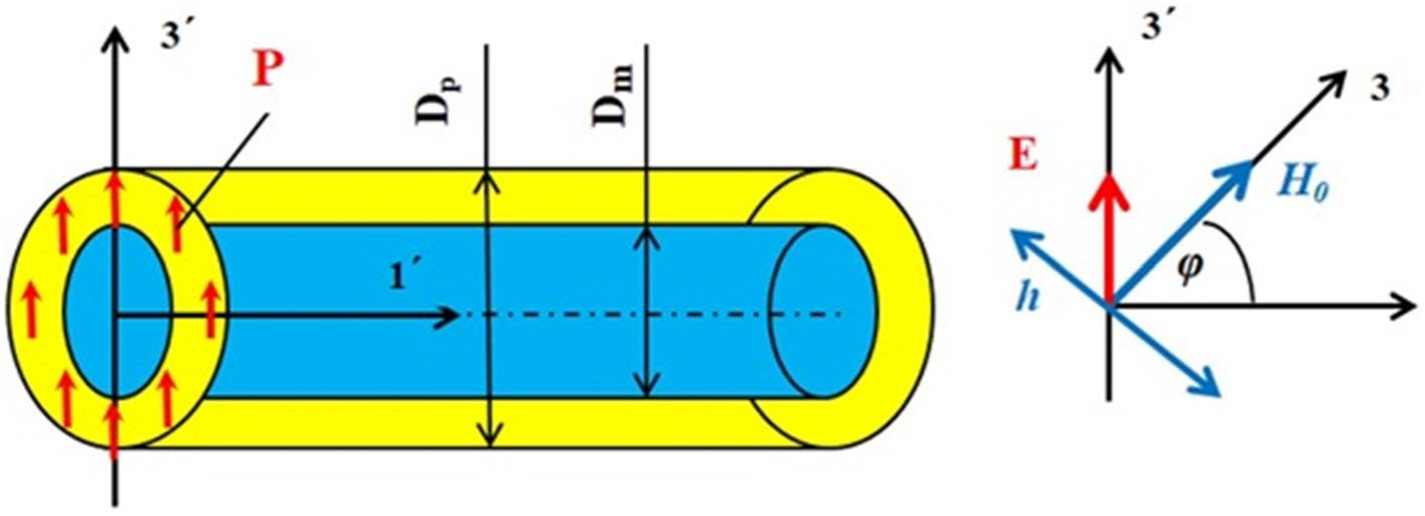
Schematic drawing of the NFO core-PZT shell structure.

A coaxial fiber as in Fig. 7 is considered. It is assumed that the length *L* along the nanofiber axis (*1′* axis) of fiber is much greater than the diameters of the ferrite core $D_{m}$ and the PZT shell $D_{p}$*.* The fiber is subjected to bias electric field ***E*** (axis *3′* in Fig. 7) perpendicular to the nanofiber axis and bias static magnetic field ***H***_***0***_ (axis *3* in Fig. 7) along the direction with an angle φ = 45° from nanofiber axis and an rf magnetic field of frequency *ω* perpendicular to bias magnetic field*.* It is assumed that both these fields are uniform throughout the sample. These field orientations correspond to the nanofiber shown in the NSMM image in Fig. 5, as well as the orientation of the magnetic field in the measurement setup. The direction of the polarization is along the bias electric field and is high enough for saturation of polarization. It follows from Fig. 2b, this assumption is well satisfied for fields E ~ 10 kV/cm.

The electric field in the piezoelectric shell will produce compressive deformations along the fiber axis and in the fiber cross-section area perpendicular to the direction of the electric field and also a tensile deformation in the cross-section area in the direction of the electric field. For calculations of these deformations it is convenient to use two coordinate systems: one coordinate system *(X*_*1*_*, X*_*2*_*, X*_*3*_*)* = *(123)* in which axis *3* coincides with the direction of the magnetic field ***H***_***0***_ and another coordinate system *(X*_*1′*_*, X*_*2′*_*, X*_*3′*_*)* = *(1′2′3′)* in which axis *3′* coincides with the direction of the electric field ***E*** (see Fig. 7). The piezoelectric deformations are small enough so that one can use the linear approximation (Hooke's law). For *E* perpendicular to the length of the nanofiber, it will cause longitudinal deformations ${}^{p}S_{{1{\prime }}}$ and deformations ${}^{p}S_{{2{\prime }}}$ and ${}^{p}S_{{3{\prime }}}$ in the plane transverse to the nanofiber axis. The system of equations for these deformations has the following form:

For PZT

2 $${}^{p}S_{{1{\prime }}} = \frac{1}{{{}^{p}Y}}({}^{p}T_{{1{\prime }}} - \nu ({}^{p}T_{{2{\prime }}} + {}^{p}T_{{3{\prime }}} ) + d_{{3{\prime }1{\prime }}} E_{{3{\prime }}} ),$$

3 $${}^{p}S_{{2{\prime }}} = \frac{1}{{{}^{p}Y}}({}^{p}T_{{2{\prime }}} - \nu ({}^{p}T_{{1{\prime }}} + {}^{p}T_{{3{\prime }}} ) + d_{{3{\prime }1{\prime }}} E_{{3{\prime }}} ),$$

4 $${}^{p}S_{{3{\prime }}} = \frac{1}{{{}^{p}Y}}({}^{p}T_{{3{\prime }}} - \nu ({}^{p}T_{{1{\prime }}} + {}^{p}T_{{2{\prime }}} ) + d_{{3{\prime }3{\prime }}} E_{{3{\prime }}} ),$$

For NFO:

5 $${}^{m}S_{{i{\prime }}} = \frac{1}{{{}^{m}Y}}({}^{m}T_{{i{\prime }}} - \nu ({}^{m}T_{{j{\prime }}} + {}^{m}T_{{k{\prime }}} )),$$

where ${}^{p}Y$, ${}^{m}Y$ are Young’s modulus of PZT and NFO respectively,$\nu$ is the Poisson’s coefficient, *T*_*λ′*_ = *T*_*i′i′*_ is the component of the stress tensor, *d*_*i′λ′*_ are the piezoelectric modules components; indices *i′ , j′, k′* = *1′, 2′, 3′* and *i′  ≠  j′  ≠  k′*, and $E_{{3{\prime }}} = \frac{V}{{D_{p} }}$ is the electric field, as a result of the applied voltage *V*, which is applied to the nanowire via electrical contact with the cantilever tip. The mechanical stress leads to change of the effective magnetic field, represented by static external field, anisotropy field, and magnetization, which collectively determine the shift of the ferromagnetic resonance. The value of this shift due to stress component ^*m*^*T*_*3*_, is given by equation^44^:

6 $$\delta H_{E} = \frac{{3\lambda_{s} }}{{M_{s} }}\,{}^{m}T_{3} ,$$

where $M_{s}$, $\lambda_{s}$ are the saturation magnetization and magnetostriction respectively, ${}^{m}T_{3}$ is the stress tensor components in ferrite core in the coordinate system in which axis *3* coincides with the direction of the magnetic field *H*_*0*_ . This stress tensor component ^*m*^*T*_*3*_ can be obtained through the components ^*p*^*T*_*i′*_*,* that are related to electric field. To find these tensor components it is convenient to use the cylindrical coordinate system *r, θ, z* with axis *z* along the nanofiber axis. Using the well-known transformation^45^, and assumption that the electric field is a small perturbation, leads to the following equation for the radial components of the strain tensor in the form^46^:

7 $$\frac{{\partial^{2} u_{r} }}{{\partial r^{2} }} + \frac{1}{r}\frac{{\partial u_{r} }}{\partial r} - \frac{{u_{r} }}{{r^{2} }} = 0.$$

The solution for Eq. (7) has the form:

8 $${}^{m}u_{r} = C_{1} r + C_{2} /r,$$

9 $${}^{p}u_{r} = C_{3} r + C_{4} /r,$$

where *C*_*1*,_ … *C*_*4*_ are integrations constants, which can be determined using the following boundary conditions: at *r* = *0* the displacement ${}^{m}u_{r} (0) = 0$; at *r* = *R*_*m*_ the displacement ${}^{m}u_{r} (R_{m}^{{}} ) = {}^{p}u_{r} (R_{m}^{{}} )$. The condition for the mechanical equilibrium on the cylindrical surface at *r* = *R*_*m*_ gives the equation:

10 $$\int\limits_{0}^{2\pi } {{}^{m}T_{rr} (R_{m}^{{}} )} d\theta = \int\limits_{0}^{2\pi } {{}^{p}T_{rr} (R_{m}^{{}} )} d\theta ,$$

and at *r* = *R*_*p*_ this condition has the form:

11 $$\int\limits_{0}^{2\pi } {{}^{p}T_{rr} (R_{p}^{{}} )} d\theta = 0.$$

Using these boundary conditions, we get for the stress tensor component *T*_*3′*_ the expression:

12 $${}^{m}T_{{3{\prime }}} = \frac{{(1 - \nu )\overline{Y}}}{{{}^{m}Y}}\frac{{(D_{p}^{2} + (1 - \nu )D_{m}^{2} )}}{{D_{p}^{2} }}{}^{m}Y(d_{{3^{\prime}3^{\prime}}} + d_{{3{\prime }1{\prime }}} )E_{{3{\prime }}} ,$$

where $\overline{Y} = ({}^{p}Y(D_{p}^{2} - D_{m}^{2} ) + {}^{m}YD_{m}^{2} )/D_{p}^{2}$ is the average value of the Young’s modulus of the NFO core-PZT shell structure.

Using the equilibrium condition for this configuration at the nanofiber’s end surfaces we get:

13 $${}^{p}T_{{1{\prime }}} ({}^{p}D^{2} - {}^{m}D^{2} ) + {}^{m}T_{{1{\prime }}} {}^{m}D^{2} = 0.$$

Using Eq. (13), we obtain the following expression for stress tensor component:

14 $${}^{m}T_{{1{\prime }}} = \frac{{{}^{p}Y}}{{\overline{Y}}}\frac{{(D_{p}^{2} - D_{m}^{2} )}}{{D_{p}^{2} }}{}^{m}Yd_{{3{\prime }1{\prime }}} E_{{3{\prime }}} .$$

The component of the stress tensor *T*_*3*_ in the *(123)* coordinate system (see Fig. 7), is obtained through the relations $x_{i}^{{}} = \beta_{{ik^{\prime}}} x_{{k{\prime }}}$, $T_{ij} = \beta_{{ik^{\prime}}} \beta_{{jl^{\prime}}} T_{{k^{\prime}l^{\prime}}}$, where $\hat{\beta }$ is the rotation matrix tensor. Using these relations, we got an expression for the stress component ${}^{m}T_{3}$ and substituting into Eq. (6) we obtained the following expression for magnetoelectric voltage constant $A_{V} = \delta H_{E} /V$:

15 $$A_{V} = \frac{{3\lambda_{s} {}^{m}Y}}{{2M_{s} D_{p} }}\left[ {\frac{{{}^{p}Y}}{{\overline{Y}}}\frac{{(D_{p}^{2} - D_{m}^{2} )}}{{D_{p}^{2} }}d_{31} + \frac{{(1 - \nu )\overline{Y}}}{{{}^{m}Y}}\frac{{(D_{p}^{2} + (1 - \nu )D_{m}^{2} )}}{{D_{p}^{2} }}(d_{{3^{\prime}3^{\prime}}} + d_{{3^{\prime}1^{\prime}}} )} \right]$$

First term in square brackets in Eq. (15) is the contribution due to compressive strains along the fiber axis, and the second term is the contribution due to compressive and tensile strains in the fiber cross-section. It is clear from Eq. (15) that the ME voltage coefficient depends on the piezoelectric constants $d_{33}$ and $d_{31}$, and diameters of the piezoelectric shell and magnetic core.

The following values were used for parameters of the structure for the numerical calculations:

$$\begin{gathered} {\text{NFO:}}{}^{m}Y = {\text{ 165 GPa}},M_{s} = {\text{ 64 kA}}/{\text{m}},\lambda s = \, - {\text{26 ppm}}, \, D_{m} = { 450}{\text{ nm}}; \hfill \\ {\text{PZT:}}{}^{p}Y = { 7}0{\text{ GPa}},d_{3^{\prime} 1^{\prime} } = - {\text{175 pm}}/{\text{V}},d_{3^{\prime} 3^{\prime} } = { 4}00{\text{ pm}}/{\text{V}},D_{p} = { 8}00{\text{ nm}}. \hfill \\ \end{gathered}$$

**Figure 8:**
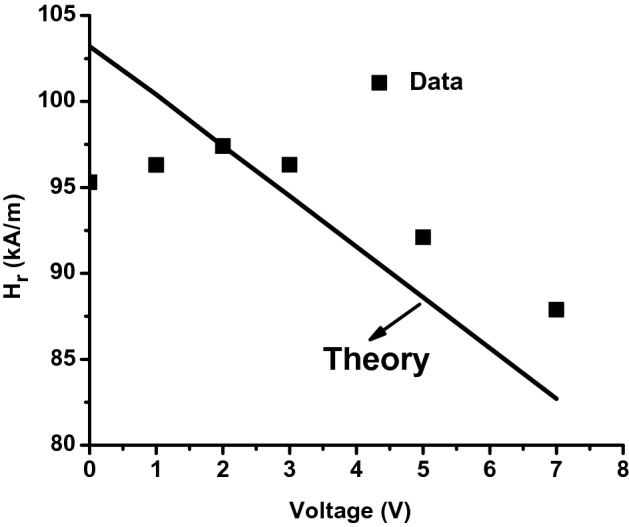
Measured (squares) and calculated (straight line) of the resonance field *H*_*r*_ at 5.4 GHz as a function of the DC voltage *V* applied to the nanofiber.

Figure 8 shows the comparison between the calculated and experimental FMR resonance field. The discrepancy between theory and experiment in the region of low voltage, 0 < V < 2 V, is possibly due to the fact that the model assumes that the polarization is directed along the electric field. It is very likely that the assumption is invalid for low voltages. A past report of significance in this regard is for the BiFeO_3_ –NiFe_2_O_4_ nanocomposites in which the electric polarization of BFO induced relatively large magnetic non-uniformity in the material^47^.

One can estimate the voltage magnetoelectric constant *A*_*V*_ from Fig. 8 and the theoretical value of *A*_*v*_ = − 2.96 kA/Vm (~ − 37 Oe/V). The corresponding estimated value of the CME coefficient *A*_*E*_ = *dH*_*r*_*/dE* = -2.36 kA/MV. The theoretical value of *A*_*v*_ is 35% higher than the measured value of 1.92 kA/m. The higher value predicted by the theory could be attributed to several factors. For estimates of *A*_*v*_ we used the values of magnetostriction and piezoelectric coupling coefficient for bulk materials. It is very likely that these parameters in nanocomposites are smaller than bulk values. One has to anticipate such a decrease since electron microscopy images (Fig. 1) for the fibers show nanocrystals rather continuous ferrite and PZT fibers.

**Figure 9:**
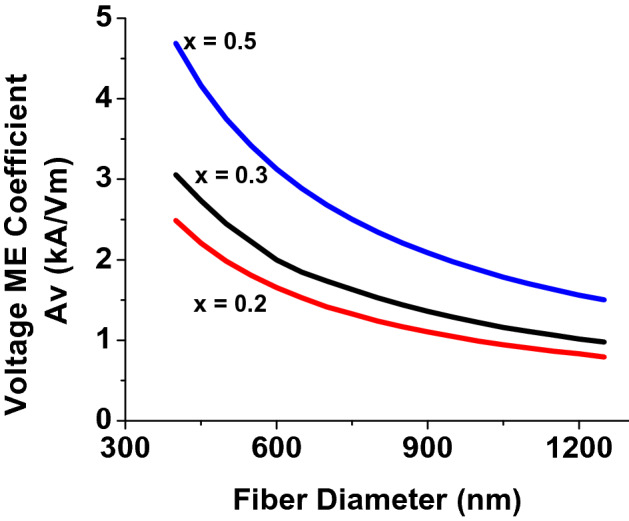
Theoretical dependence of the voltage ME coefficient on the outer diameter of the core–shell fiber. Results are for a series of the ratio of the diameter of the ferrite core to the outer diameter of the PZT shell.

The theory was extended to include results on the dependence of the voltage ME coefficient on the fiber diameter for a series of ratio of diameter of the ferrite core to diameter of PZT shell. Figure 9 shows estimated voltage-ME coefficient *A*_*v*_ as a function of the outer diameter of the fiber. Estimates are shown for a series of *x* = *Dm/Dp* where *D*_*m*_ is the diameter of the magnetostrictive core and *D*_*p*_ is outer diameter of the piezoelectric shell. The model predicts a significant increase in A_v_ with decreasing fiber diameter. Therefore, it is desirable to synthesize fibers with smallest possible diameter to achieve strong magnetoelectric coupling.

There have been no reports so far on the measurements of CME effects either in an individual ferrite-ferroelectric fiber or in films of fibers. The effect, however, was investigated in a variety of ferromagnetic-ferroelectric thick film and thin film composites. Since the parameter of importance for voltage tunable ferrite devices is the voltage-ME coefficient *A*_*v*_ and most of the reports in the past expressed *A*_*v*_ in units of Oe/V, we compare the *A*_*v*_-values in units of Oe/V and list the SI units in parenthesis. The CME effects in several ferrimagnetic-ferroelectric composites systems were studied by voltage tunable FMR measurements. These include yttrium iron garnet (YIG), nickel ferrite (NFO), M-type strontium (SrM) or barium (BaM) hexagonal ferrites for the magnetic phase and PZT or lead magnesium niobite- lead titanate (PMN-PT) for the ferroelectric phase^3,21,48–54^. Since YIG and M-type hexaferrites have weak magnetostriction, *A*_v_-values in the range 0.005–0.01 Oe/V (0.4–0.8 A/mV) were reported in composites with PZT or PMN-PT ^50,51^. In composites of PZT or PMN-PT and a micron-thick NFO prepared by chemical vapor deposition techniques *A*_*v*_ -values were in the range 0.01–0.08 Oe/V (0.8–6.4 A/mV)^49^. For Fe_3_O_4_ films deposited directly onto ferroelectrics *A*_*v*_*-*values as high as 0.25 Oe/V (20 A/mV) were reported^52^. Composites with films of the ferromagnetic alloy FeGaB and PZT, PMN-PT or PZN-PT were studied specifically for applications in tunable inductors and were found to show strong ME coupling^54^. Consequently, the CME coefficient for NFO-PZT core–shell nanofiber is one of the highest values reported for ferrite-ferroelectric systems.

## Conclusions

Coaxial nanofibers of NFO and PZT were successfully synthesized by electrospinning. The microstructure of the core and shell was characterized using SEM and NSMM. The converse ME coefficient was investigated by applying a DC voltage *V* to the fibers and subsequent measurements of the resulting shift in the FMR as a function of *V*. The voltage-CME coefficient of 24 Oe/V (1.92 kA/m) was determined from the FMR data. A theoretical model for the effect is developed and the estimated *A*_*v*_ of 37 V/Oe (2.96 kA/V) is in general agreement with the experimental value. Thus, the CME coefficient reported here is one of the highest for any ferrite-ferroelectric composite system.

## Methods

Multiferroic ME material composites are known to use several ferroelectrics, including barium titanate, lead zirconate titanate (PZT), or lead magnesium niobate-lead titanate (PMN-PT), and ferromagnetic/ferrimagnetic oxides, metals or alloys^4,5^. The core–shell fiber of ferrite NiFe_2_O_4_ (NFO) and ferroelectric PbZr_0.58_Ti_0.42_O_3_ (PZT) used in this study were synthesized by electrospinning^39^. The synthesis details are provided in Ref. 26. The process involved preparation of sol for the two oxides that were loaded on to a dual syringe pumping system and the core–shell fibers obtained by dispensing the sols through a stainless-steel coaxial needle under an electric field of 1.5–2 kV/cm. The fibers were collected on a rotating aluminum drum. The fibers were dried at 40 ℃ for 24 h and annealed in air at 650 ℃ for 1 h. An X ray diffractometer (XRD), a scanning electron microscope (SEM) and a near-field scanning microwave microscope (NSMM) were used to characterize the phase contents and structure for the core–shell fibers. Magnetic and ferroelectric measurements were carried out using a Faraday balance and a ferroelectric test system (Radiant Technologies, Inc.). (Certain commercial equipment, instruments, or materials are identified in this paper in order to specify the experimental procedure adequately. Such identification is not intended to imply recommendation or endorsement by the National Institute of Standards and Technology, nor is it intended to imply that the materials or equipment identified are necessarily the best available for the purpose).
